# Peroxisome Proliferator-Activated Receptor-γ Agonist 15d-Prostaglandin J_2_ Mediates Neuronal Autophagy after Cerebral Ischemia-Reperfusion Injury

**DOI:** 10.1371/journal.pone.0055080

**Published:** 2013-01-25

**Authors:** Feng Xu, Jian Li, Wei Ni, Yi-wen Shen, Xiao-ping Zhang

**Affiliations:** 1 Department of Neurosurgery, Huashan Hospital, Shanghai Medical College, Fudan University, Shanghai, China; 2 Department of Neurosurgery, Huadong Hospital, Shanghai Medical College, Fudan University, Shanghai, China; 3 Department of Nuclear Medicine, Shanghai 10^th^ People’s Hospital, Tongji University School of Medicine, Shanghai, China; UAE University, Faculty of Medicine & Health Sciences, United Arab Emirates

## Abstract

Peroxisome proliferator-activated receptor-γ (PPAR-γ) has recently emerged as potential therapeutic agents for cerebral ischemia-reperfusion (I/R) injury because of anti-neuronal apoptotic actions. However, whether PPAR-γ activation mediates neuronal autophagy in such conditions remains unclear. Therefore, in this study, we investigated the role of PPAR-γ agonist 15-PGJ_2_ on neuronal autophagy induced by I/R. The expression of autophagic-related protein in ischemic cortex such as LC3-II, Beclin 1, cathepsin-B and LAMP1 increased significantly after cerebral I/R injury. Furthermore, increased punctate LC3 labeling and cathepsin-B staining occurred in neurons. Treatment with PPAR-γ agonist 15d-PGJ_2_ decreased not only autophagic-related protein expression in ischemic cortex, but also immunoreactivity of LC3 and cathepsin-B in neurons. Autophagic inhibitor 3-methyladenine (3-MA) decreased LC3-II levels, reduced the infarct volume, and mimicked some protective effect of 15d-PGJ_2_ against cerebral I/R injury. These results indicate that PPAR-γ agonist 15d-PGJ_2_ exerts neuroprotection by inhibiting neuronal autophagy after cerebral I/R injury. Although the molecular mechanisms underlying PPAR-γ agonist in mediating neuronal autophagy remain to be determined, neuronal autophagy may be a new target for PPAR-γ agonist treatment in cerebral I/R injury.

## Introduction

Restoration of blood flow following ischemic stroke plays a critical role in tissue repair and functional recovery. However, after a period of ischemia, reperfusion may exacerbate the injury initially caused by ischemia, producing a so-called “cerebral ischemia-reperfusion (I/R) injury”. Multiple pathological processes are involved in ischemic neuronal damage, including energy metabolism disturbance, excitotoxicity, oxidative stress, inflammation, necrotic and apoptotic cell death. Despite of growing understanding of the mechanisms of neuronal death accompanying cerebral I/R, effective therapy has remained elusive.

Peroxisome proliferator-activated receptor-γ (PPAR-γ) is a ligand-activated transcription factor belonging to nuclear hormone receptor superfamily. Structurally diverse ligands activate PPAR-γ, including 15-deoxy-△^12,14^-prostagladlin J_2_ (15d-PGJ_2_) [1], lysophosphatidic acid [2], nitrolinoleic acid [3], as well as the synthetic thiazolidinedione (TZD) class of antidiabetic drugs such as troglitazone, ciglitazone, pioglitazone, and rosiglitazone [4]. PPAR-γ agonists have been shown to protect against cerebral infarction in a rat I/R stroke model [5]–[8]. These neuroprotective effects have been related to antioxidative actions and inhibition of inflammation. Recent studies demonstrated the anti-neuronal apoptotic actions of PPAR-γ against cerebral I/R through inhibiting caspase 9 and caspase 3 activation [9], [10]. However, types of neuronal cell death induced by cerebral I/R include not only apoptosis, but also autophagy, characterized by numerous autophagic vacuoles. Increasing evidence has shown an involvement of enhanced autophagy in neuronal death following cerebral ischemia [11]–[23]. Moreover, activated autophagy contributes to ischemic neuronal injury after cerebral I/R injury [22], [23]. Recently, PPAR-γ activation has been shown to be associated with autophagy in cancer cells [24]. However, it is unclear whether PPAR-γ agonist mediates neuronal autophagy after cerebral I/R injury. Therefore, further studies focused on neuronal autophagy may provide a potential target for PPAR-γ agonist treatment in cerebral ischemia.

In the present study, we investigated the role of PPAR-γ agonist 15-PJG_2_ on neuronal autophagy induced by I/R. Our results showed the involvement of neuronal autophagy after cerebral I/R injury. Moreover, we showed for the first time that PPAR-γ agonist 15d-PGJ_2_ inhibits neuronal autophagy after cerebral I/R. Furthermore, inhibition of autophagy might play a role in neuroprotection against cerebral injury by 15d-PGJ_2._

## Materials and Methods

### Animal Models

Male ICR mice (body weight 25–30 g) were purchased from the Center for Experimental Animals of Fudan University. All the procedurals were carried out in strict accordance with the recommendations in the Guide for Care and Use of Laboratory Animals of the National Institutes of Health. The protocol was approved by the Committee on the Ethics of Animal Experiments of Fudan University. Focal cerebral ischemia and reperfusion (I/R) models were induced using the suture occlusion technique [25]. After the mice were deeply anesthetized with isoflurane (2%), the right common carotid artery (CCA), external carotid artery (ECA) and internal carotid artery (ICA) were surgically exposed. The external carotid artery was then isolated and coagulated. A 6–0 nylon suture with silicon coating (Doccol Corporation, Redlands, USA) was inserted into the internal carotid artery through the external carotid artery stump and gently advanced to occlude the middle cerebral artery (MCA). Laser-Doppler flowmetry (LDF, ML191 Laser Doppler Blood FlowMeter, Australia) was used to monitor the blockade of cerebral blood flow of middle cerebral artery territory. After 2 h of MCA occlusion (MCAO), the suture was carefully removed to restore blood flow (reperfusion), the neck incision was closed, and the mice were allowed to recover. Those animals recovered blood flow to 80% of pre-ischemia levers were used for further study. The body temperature was carefully monitored during the post-operation period and until complete recovery of the animal from the anesthetic. Sham animals underwent identical surgery but the suture was not inserted.

Intracerebroventricular (icv) injections were performed in the right lateral ventricle with 10 µL of 15d-PGJ_2_ (1 to 50 pg) at a rate of 2 µL/min. The following coordinates: Anterior, 0.5 mm caudal to bregma; Right, 1.0 lateral to midline; and Ventral, 2.5 mm ventral to dural surface. 3-methyladenine (3-MA) was obtained from Sigma (St. Louis, MO). Icv injections were performed in the ipsilateral ventricle with 2 µL of a 30 mg/ml solution prepared in saline (0.9% NaCl). Mice were injected immediately before reperfusion.

### Evaluation of Infarct Volume and Motor Deficits

After 24 h of reperfusion, animals were anesthetized with intraperitoneal injection of 4% choral hydrate, the brains were dissected and sliced in a plastic module (Havard Apparatus, Holliston, USA), five sections of 1.5 mm thickness were made and stained with 2% 2,3,5-triphenyltetrazolium chloride(TTC) for 30 min and then fixed with 4% paraformaldehyde. Lesioned areas not stained red with TTC were quantitatively analyzed with Sigma Scan Pro 5. Infarct volume was calculated using slice thickness and the measured areas of lesion, expressed as a percentage of ipsilateral hemisphere [25].

The motor deficits in mouse subjected to MCAO were evaluated by an examiner without knowing experimental conditions using the scales as previously described. 0 point, mice behave normally; 1 point, mice cannot fully stretch their left front legs; 2 points, mice turn around into a circle; 3 points, mice fall down to the left side; 4 points, mice cannot move by themselves, losing their consciousness.

### Transmission Electron Microscopy

After various time points (0, 6, 12, and 24 h post reperfusion), mice were perfused with PBS (pH 7.4) followed by precooled PBS containing 4% paraformaldehyde and 2.5% glutaraldehyde. The cortex in the ischemic core area were rapidly isolated, and were cut into small sections and kept in 2.5% glutaraldehyde in 0.1 mol/L PBS (pH 7.4). The sections were postfixed in 1% osmium tetroxide for 1 h, dehydrated in graded ethanol, and embedded in epoxy resin. Polymerization was performed at 80°C for 24 h. Blocks were cut on a Reichert ultramicrotome into ultrathin sections (60–70 nm), which were poststained with uranylacetate and lead citrate, and viewed under a Hitachi 7100 electron microscopy (Nikon).

### Western Blot

Western blotting was used to analyze protein expression in the ipsilateral ischemic cortex. In brief, samples were homogenized in lysis buffer (150 mM NaCl, 1% SDS, 1% Triton, 1% Na-deoxycholate, 1 mmol/L EDTA, 50 mmol/L Tris-Cl pH 7.4, and protease inhibitor cocktail) (Roche, Basel, Switzerland). Protein concentrations were determined using a BCA kit (Piece, Rockford, IL, USA). Samples were boiled in SDS-PAGE loading buffer for 5 min and were analyzed by loading equivalent amounts of total proteins (30 µg) onto 10% SDS-polyacrylamide gels. Proteins were subsequently transferred to a nitrocellulose membrane, which was then incubated with 5% skimmed milk in Tris–buffered saline with 0.1% Tween 20 (TBST) for 1 h at room temperature. Afterward, the membranes were incubated with the primary antibodies against LC3 (Abcam, 1∶1000); Beclin1 (Santa Cruz, 1∶500); cathepsin-B (Santa Cruz, 1∶500); LAMP1 (Abcam, 1/500); PPAR-γ (Santa Cruz, 1∶500); and β-actin (Sigma, 1∶5000) overnight at 4°C. After washing with TBST, membranes were then incubated with fluorescence secondary antibodies (LI-COR Biosciences, Lincoln, Nebraska USA), and the signal was read with an Odyssey® Western Blot Analysis system (LI-COR Biosciences, Lincoln, Nebraska USA). The signal intensity of primary antibody binding was quantitatively analyzed with Sigma Scan Pro 5 and was normalized to a loading control β-actin.

### Immunofluorescence

Mice were anesthetized with 4% chloral hydrate and transcardially perfused with PBS (pH 7.4) followed by PBS containing 4% paraformaldehyde (pH 7.4). Perfusion-fixed brains were post-fixed in PBS containing 4% paraformaldehyde overnight. Coronal brain sections (10 µm thick) were cut with a cryostat. For immuofluorescence labeling, the sections were precubated for 30 minutes in 0.3% Triton X-100 in PBS, and then incubated overnight at 4°C with the primary antibody in 1% bovine serum albumin, 0.1% Triton X-100 in 0.1 M PBS. After rinsing three times with PBS, sections were incubated for 2 h in fluorochrome-coupled secondary antibody (Alexa 488 or Alexa 568, Molecular Probes, Eugene) respectively. After rinsing with PBS, the sections were mounted with a Zeiss confocal laser-scanning microscope. The antibodies used were as follows: anti-NeuN (MAB377, 1∶200); anti-LC3 (Abcam, 1∶200); cathepsin-B (Abcam, 1∶200).

### Statistical Analysis

Statistical analysis was performed with one-way ANOVA followed by Dunnett t-test. p<0.05 was considered to be significant.

## Results

### Involvement of Neuronal Autophagy after Cerebral I/R Injury

**Figure 1:**
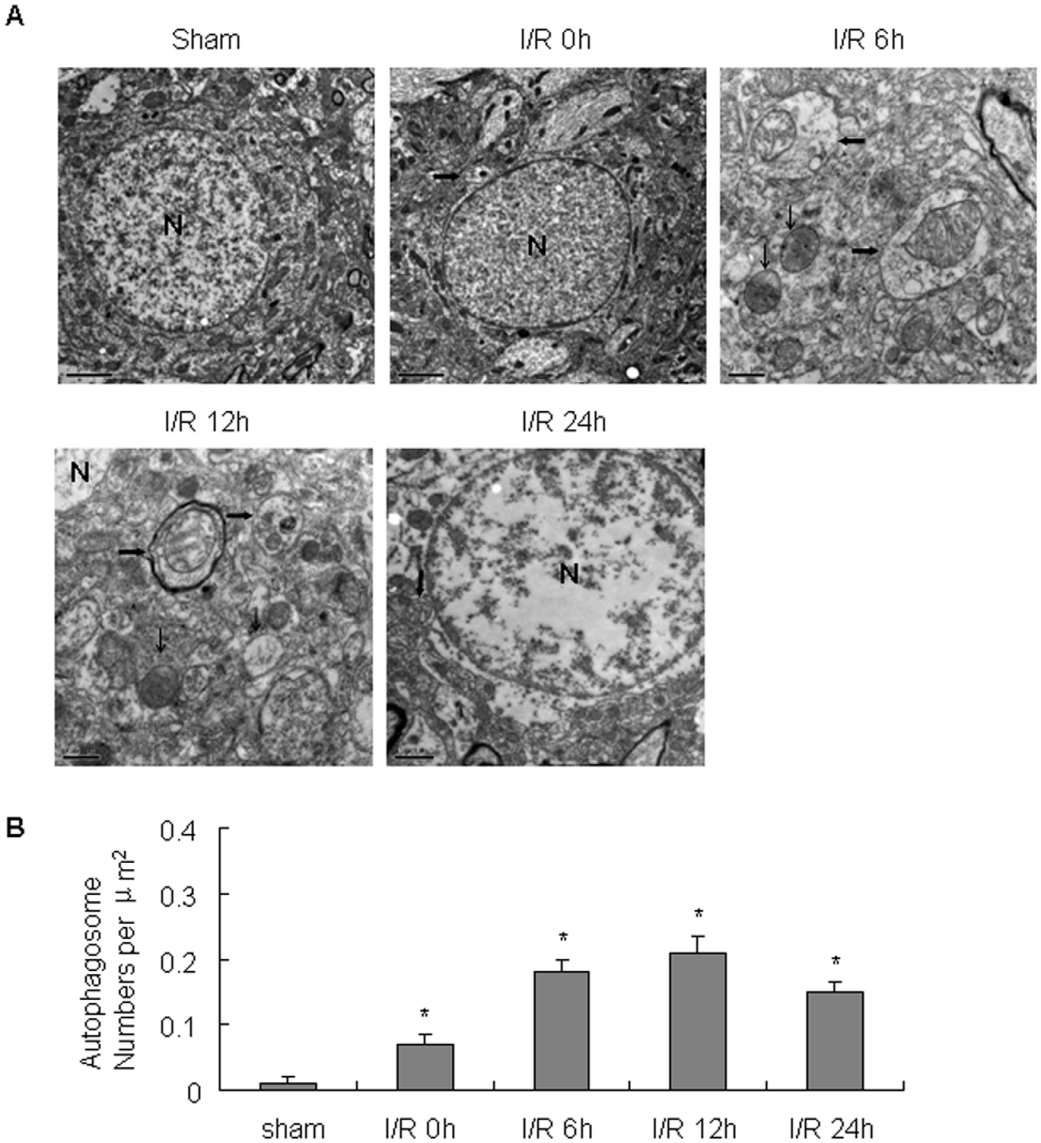
Electron micrographs of morphological changes of cortical neurons after cerebral I/R injury. (A) N, nucleus; Broad arrows represent autophagosomes; Narrow arrows represent mitochondria. (B) Quantitative analysis of the nubmeber of autophagosomes. Three animals in each group and 10 fields for each animal were examined. *p<0.05 versus sham group.

To evaluate the involvement of neuronal autophagy after cerebral I/R injury, we used transmission electron microscopy, Western blots, and immunohistochemistry. Transmission electron microscopy was used to examine morphology changes of neurons 0 to 24 h after cerebral I/R injury. Cortical neurons from sham-operated control mice contained normal appearance of nucleus, rough endoplasmic reticulum (ER), Golgi apparatus, mitochondria, and lysosomes. In contrast, cortical neurons subjected to I/R injury displayed an increase in the number of autophagosomes (APs) and autolysosomes (ALs). APs were identified as bubble-like vacuoles enclosing recognizable cytoplasmic structures (Fig. 1).

**Figure 2:**
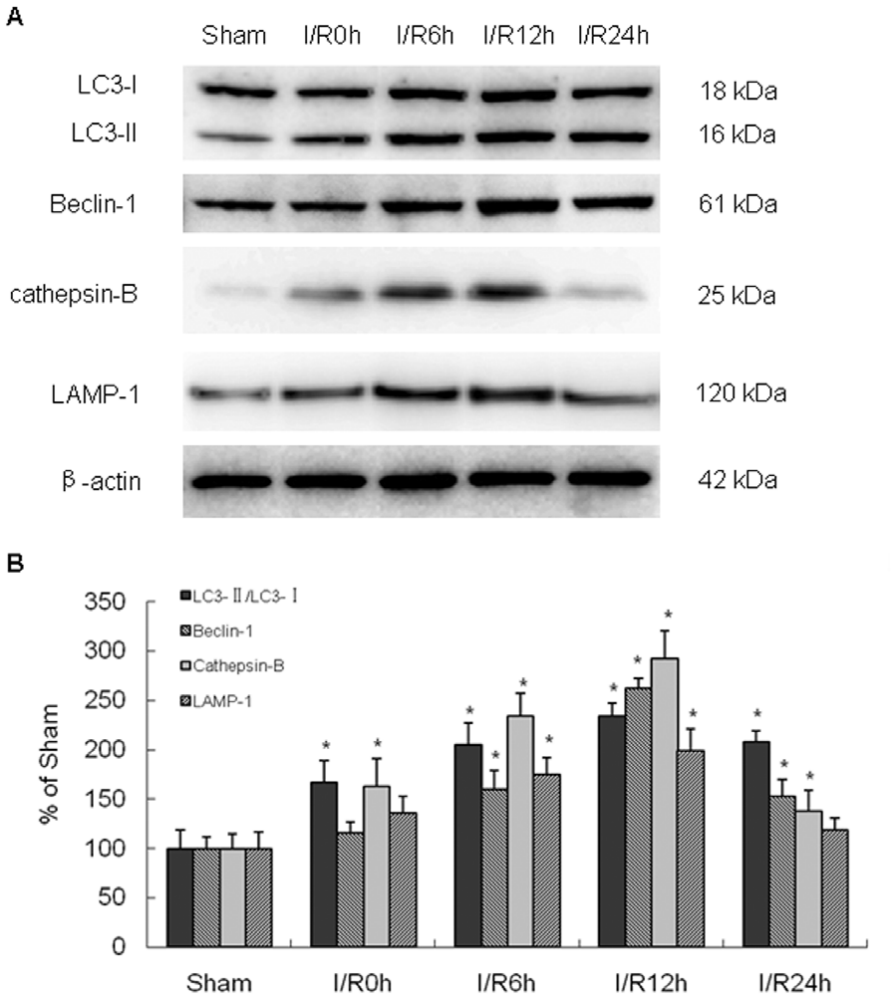
Western Blot analysis of autophagic-related protein expression after cerebral I/R injury. The level of LC3-II and Beclin 1 in ischemic cortex increased significantly from 6 to 24 h after reperfusion, with a maximal induction at 12 h. The expression of active cathepsin-B and LAMP1 in ischemic cortex increased significantly from 6 to 12 h after reperfusion, with a maximal induction at 12 h. Optical density of respective protein bands were analyzed with Sigma Scan Pro 5 and normalized to the loading control (β-actin). *p<0.05 versus sham group.

Western blots were performed to examine autophagic-related protein expression at 0 to 24 h after I/R. Cytoplasmic form LC3 (LC3-I) is diffusely distributed in the cytoplasm, but modified and conjugated to a phosphatidylethanolamine (PE) leading to lipidated form (LC3-II), which is attached to the autophagosome membrane during autophagy activation. LC3-II is the most widely used marker for revealing the presence of autophagosomes. Another marker, Beclin 1, belongs to the class 3 phosphoinositide 3-kinase (PI3K) complex and is involved in the early stages of autophagosome formation. As shown in Fig. 2, the expression of LC3-II in ischemic cortex increased significantly from 6 to 24 h after reperfusion, with a maximal induction at 12 h. Beclin 1 levels were also significantly upregulated and peaked at 12 h.

To determine whether the increase in auophagosomes reflected an increase in autophagic flux or a defect in lysosomal fuction, we also investigated the activity of the lysosomal pathway. Therefore, we performed western blots against the lysosomal protease cathepsin-B, and the lysosomal-associated membrane protein 1 (LAMP1). As shown in Fig. 2, active cathepsin-B expression in ischemic cortex increased significantly from 6 to 12 h after reperfusion, with a maximal induction at 12 h. LAMP1 levels were also significantly upregulated after I/R, which was coincident with the increased levels of cathepsin-B.

**Figure 3:**
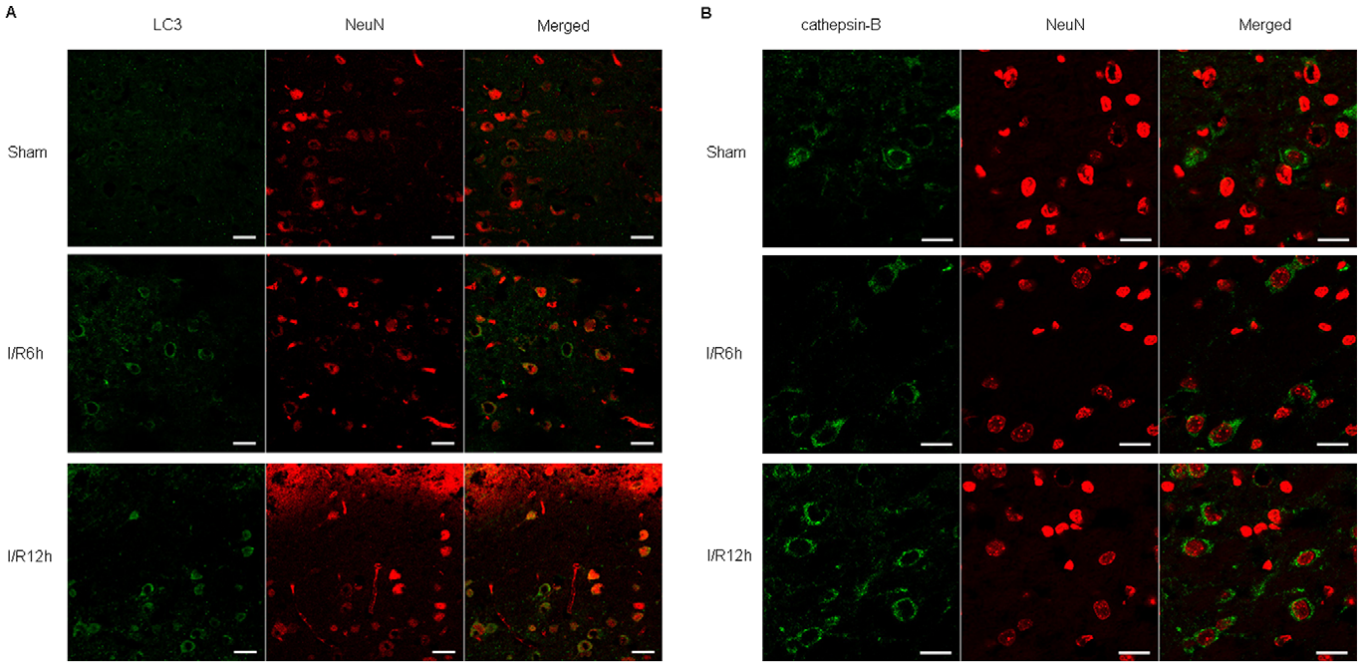
Immunohistochemistry for LC3 and cathepsin B in neurons after cerebral I/R injury. (A) In sham-operated animals, cortical cells displayed diffuse and weak staining for LC3 in the cytosol. After I/R, intense LC3 staining appeared granular in the cytosol of cortical cells. Double staining for LC3 (green) and NeuN (red) showed that increase in LC3 punctate labeling occurred in cortical neurons. (B) In sham-operated animals, cortical cells displayed fine, granular, and perinuclear cathepsin-B staining. After I/R, cathepin-B granules became progressively larger and irregular, and the granular pattern was finally replaced with diffuse cytoplasmic staining. Double staining for cathepsin-B (green) and NeuN (red) showed that increased expression of cathepsin-B occurred mainly in neurons. Bar = 20 µm.

Immunohistochemistry was performed to examine LC3 and cathepsin-B immunoreactivity at 6 and 12 h after I/R. In sham-operated animals, cortical cells displayed diffuse and weak staining for LC3 in the cytosol. After I/R, intense LC3 staining appeared granular in the cytosol of cortical cells. Double staining for LC3 and the neuronal marker Neuronal Nuclei (NeuN) showed that increase in LC3 punctate labeling occurred in cortical neurons (Fig. 3A). This tendency was also consistent with cathepsin-B staining. In sham-operated animals, cortical cells displayed fine, granular, and perinuclear cathepsin-B staining. After I/R, cathepsin-B granules became progressively larger and irregular, and the granular pattern was finally replaced with diffuse cytoplasmic staining (Fig. 3B). Double staining for cathepsin-B and NeuN showed that increased expression of cathepsin-B occurred mainly in neurons.

### PPAR-γ Agonist 15d-PGJ_2_ Inhibits Neuronal Autophagy after Cerebral I/R Injury

**Figure 4:**
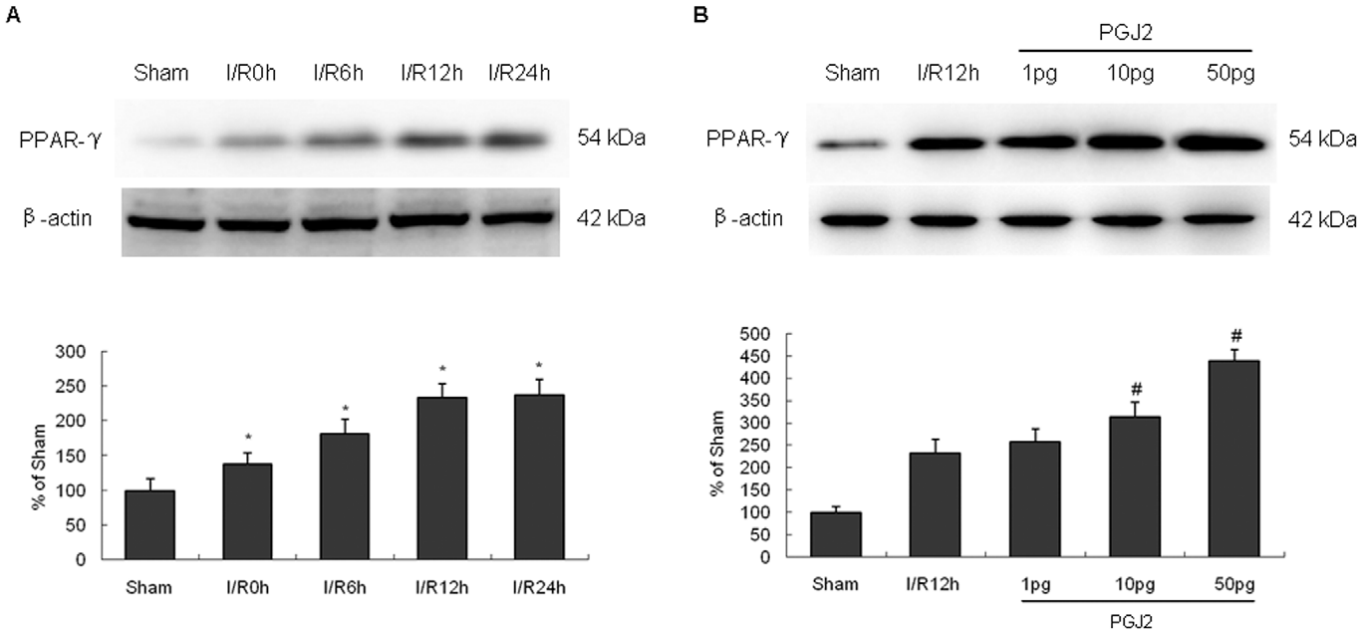
PPAR-γ protein expression in cortex after cerebral I/R injury. (A) The level of PPAR-γ in ischemic cortex was higher than control in a time-dependent manner. (B) 15-PGJ_2_ upregulated PPAR-γ expression in ischemic cortex in a concentration-dependent manner. *p<0.05 versus sham group, ^#^p<0.05 versus I/R group.

To determine whether PPAR-γ agonist 15d-PGJ_2_ mediates neuronal autophagy after cerebral I/R injury, we used intraventricular injection of 15-PGJ_2_ (1 to 50 pg) immediately before reperfusion. First, we first examined the PPAR-γ protein expression in cortex after cerebral I/R injury. The PPAR-γ protein level in ischemic cortex was higher than control in a time-dependent manner. Moreover, 15-PGJ_2_ upregulated PPAR-γ expression in ischemic cortex in a concentration-dependent manner (Fig. 4).

**Figure 5:**
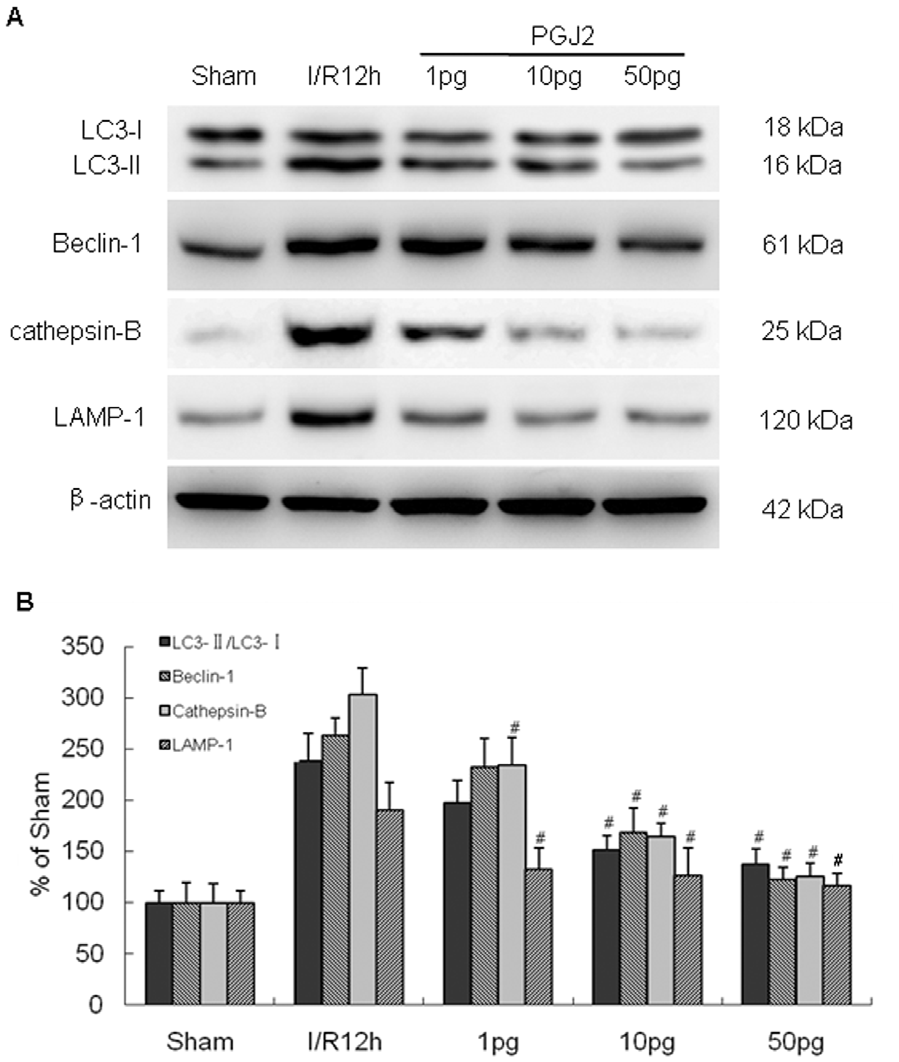
Effect of 15-PGJ_2_ treatment on autophagic-related protein expression in ischemic cortex after cerebral I/R injury. The expression of LC3-II, Beclin 1, cathepisin-B, and LAMP1 expression significantly increased at 12 h after reperfusion. Treatment with 15-PGJ_2_ at 1 to 50 pg significantly decreased LC3-II, Beclin 1, cathepisin-B, and LAMP1 expression after I/R injury. Optical density of respective protein bands were analyzed with Sigma Scan Pro 5 and normalized to the loading control (β-actin). ^#^p<0.05 versus I/R group.

Then, we analyzed the expression of several autophagic-related proteins in ischemic cortex at 12 h after I/R injury by western blot. I/R injury resulted in a significant increase of LC3-II, Beclin 1, cathepsin-B, and LAMP1 expression compared with sham-operated group (Fig. 5). Treatment with 15-PGJ2 at 1 to 50 pg significantly decreased LC3-II, Beclin 1, cathepsin-B, and LAMP1 expression after I/R injury. The maximal reduction occurred at 50 pg, then we used 50 pg of 15-PGJ2 in the remaining studies.

**Figure 6:**
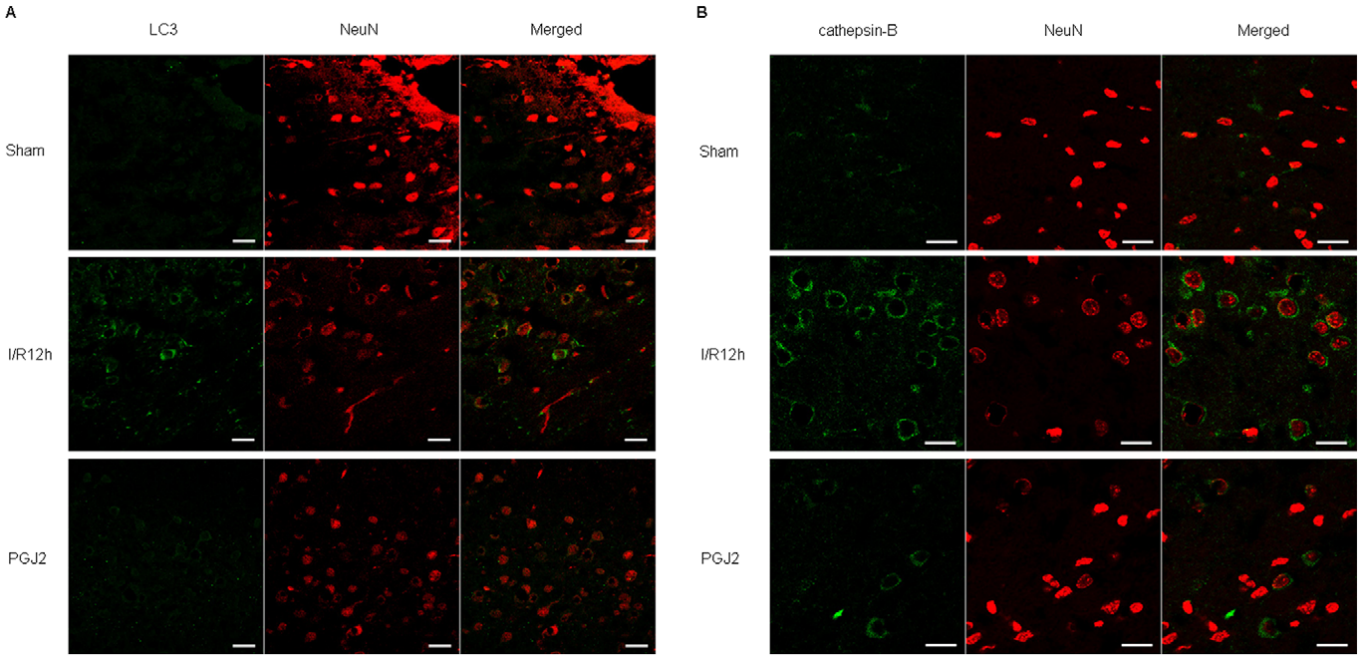
Effect of 15-PGJ_2_ treatment on LC3 and cathepsin-B immunoreactivity after cerebral I/R injury. Double staining showed that LC3 punctate labeling and cathepsin-B immunoreactivity increased in neurons compared with sham-operated animals at 12 h after I/R injury. 15-PGJ2 at 50 pg effectively blocked the activation of autophagy as evidence by inhibiting immunoreactivity of LC3 (A) and cathepsin-B (B). Bar = 20 µm.

To confirm the effect of PPAR-γ agonists on neuronal autophagy, we also performed immunohistochemistry to examine LC3 and cathepsin-B immunoreactivity at 12 h after I/R. Double staining showed that I/R injury increased LC3 punctate labeling and cathepsin-B immunoreactivity in neurons compared with sham-operated animals. 15-PGJ_2_ at 50 pg effectively blocked the activation of autophagy as evidence by inhibiting immunoreactivity of LC3 and cathepsin-B (Fig. 6). These results indicate that PPAR-γ agonist 15d-PGJ_2_ inhibits neuronal autophagy after cerebral I/R injury.

### Inhibition of Autophagy Plays a Role in 15d-PGJ_2_ Neuroprotective Effects Against Cerebral I/R Injury

**Figure 7:**
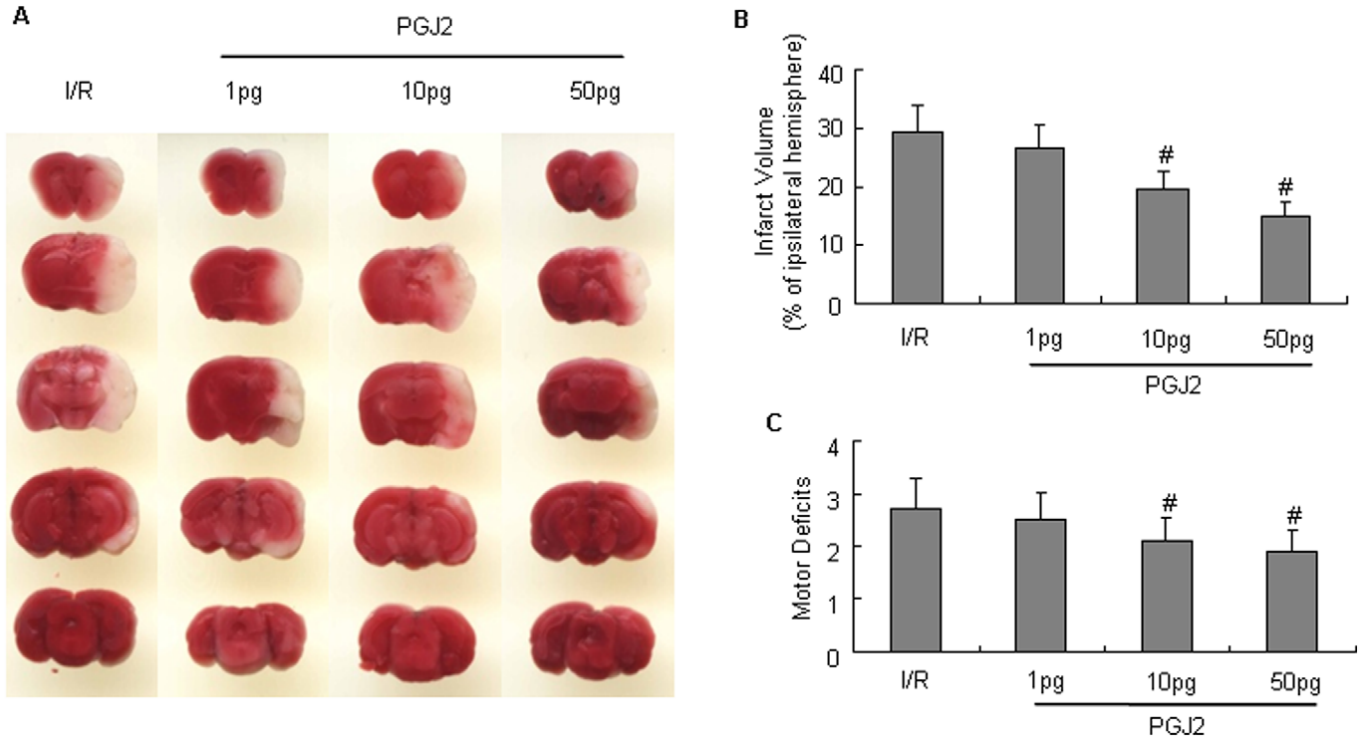
Neuroprotective effects of 15-PGJ_2_ against cerebral I/R injury. 15-PGJ_2_ (1, 10, 50 pg) was administered icv immediately before reperfusion. (A) Five consecutive TTC-stained coronal brain slices arranged in cranial to caudal order 24 h after I/R. The white brain area represents infracted tissue. Infarct volume (B), neurological deficits (C) was measured 24 h after I/R. ^#^p<0.05 versus I/R group.

To investigate the neuroprotective effects of 15d-PGJ_2_ against cerebral I/R injury, 15-PGJ_2_ (1, 10, 50 pg) was administered icv immediately before reperfusion, and infarct volumes were assessed 24 h after reperfusion. No infarction was observed in sham-operated group. Extensive infarction was detected in cerebral cortical and subcortical areas in mice subjected to I/R (Fig. 7A). 15d-PGJ_2_ (10 and 50 pg) reduced infarct volume by 33.8% and 49.5%, respectively (Fig. 7B). Neurological deficits were examined and scored on a four-point scale. Mice subjected to I/R showed significant motor behavior deficits. 15d-PGJ_2_ (10 and 50 pg) showed a significant reduction in ischemia-induced neurological deficits (Fig. 7C).

**Figure 8:**
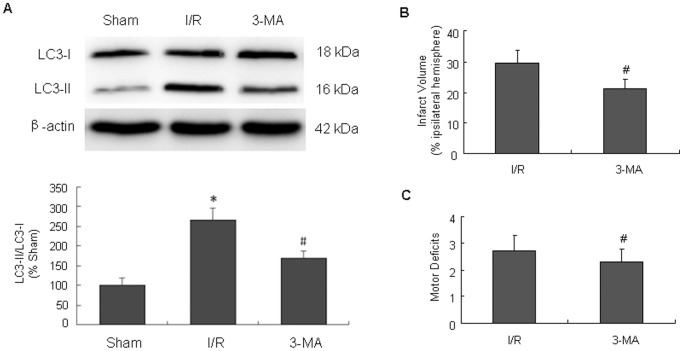
Protective effects of 3-mehtyladenine (3-MA) following cerebral I/R injury. 3-MA (60 µg) solutions were injected icv immediately before reperfusion. (A) The changes of LC3 after the treatment of 3-MA. 3-MA significantly decreased LC3-II levels at 24 h after I/R. Effect of 3-MA on infarct volumes (B) and neurological deficits (C). 3-MA significantly reduced the infarct volume, and ameliorate the neurological symptoms at 24 h after I/R. ^#^p<0.05 versus I/R group.

To conclude if inhibition of autophagy plays a role in the neuroprotection of 15d-PGJ_2_, we use 3-MA to investigate whether autophagy inhibitors produce similar effects. First, we examined the effect of 3-MA on the protein levels of LC3. Western blot analysis showed that icv injection of 3-MA (60 µg) significantly decreased LC3-II levels at 24 h after I/R injury (Fig. 8A). Then, we examined whether the autophagy inhibitor could provide neuoprotection against I/R injury. 3-MA (60 µg) administered before reperfusion significantly reduced the infarct volume, and ameliorate the neurological symptoms compared to I/R group (Fig. 8B, C). These results implicated that 3-MA could inhibit autophagy activation and ameliorate the ischemic injury. Taken together, inhibition of autophagy might play a role in 15d-PGJ_2_ neuroprotective effects against cerebral I/R injury.

## Discussion

Autophagy is a highly regulated and evolutionary conserved process for bulk degradation and recycling of cytosolic components, such as long-lived proteins and organelles [26]–[28]. In neurons, constitutively active autophagy at lower levels is important for maintaining homeostasis and protein quality control under normal conditions [29]. Inadequate or defective autophagy, rather than excessive autophagy, promotes neuronal cell death [29]–[32]. Recently, increased autophagy has been reported in cerebral ischemia injury, including hypoxia-ischemia (HI) [11]–[16], global [17]–[19] and focal ischemia [20]–[23]. Rami et al. showed a dramatic elevation in Beclin 1 levels in neurons in the penumbra of adult rats after focal cerebral I/R. The authors also showed that all Beclin 1 upregulating cells display dense staining of LC3 [21]. Liu et al. showed that LC3-II protein was upregulated in post-ischemic brain tissues after global cerebral I/R [18]. Consistent with previous studies, our results also demonstrated that the expression of LC3-II and Beclin 1 in ischemic cortex increased significantly from 6 to 24 h after focal cerebral I/R, with a maximal induction at 12 h. Furthermore, immunohistochemistry analysis showed an increase in LC3 punctate labeling in ischemic neurons from 6 h. These data suggest the involvement of enhanced autophagy in neuronal death following focal cerebral I/R. However, this increase might be due to a defect in lysosomal function causing an accumulation of autophagosomes, or a real increase in autophagic flux. Therefore, we also investigated the activity of the lysosomal pathway after focal cerebral I/R.

Previous studies have demonstrated that lysosomal and autophagic activities are increased in the ischemic neurons after cerebral HI, transient or permanent focal cerebral ischemia [16], [20], [22]. Ginet et al. showed that neonatal cerebral HI increased lysosomal activities including cathepsin D and LAMP1 in cortical-damaged neurons [16]. Puyal et al. showed that transient middle cerebral artery occlusion (MCAO) increased the numbers of cathepsin D, LAMP1-positive neurons in neonatal rats. Moreover, double labeling showed that the strong punctuate autophagosomal labeling (LC3) and the strong lysosomal labeling (cathespin D and LAMP1) are in the same neurons [22]. Wen et al. showed the protein levels of cathepsin B increased in ischemic cortex after permanent MCAO [20]. Consistent with previous studies, our results demonstrated that active cathepsin-B and LAMP1 expression increased significantly from 6 to 12 h after focal cerebral I/R, with a maximal induction at 12 h. Furthermore, immunohistochemistry analysis showed increased expression of cathepsin-B occurred mainly in neurons. These data demonstrated an enhancement of autophagic flux following focal cerebral I/R. However, Liu et al. put forward different opinions that the accumulation of protein aggregate-associated organelles following global cerebral ischemia is likely to be because of failure of the autophagic pathway, as result of lysosome deficiency [18]. This discrepancy may result from different ischemic models, different evaluation methods or different observing time.

Although the role of autophagy in neuronal death is still debating, increasing evidence suggests that autophagy activation contributes to ischemic neuronal injury after cerebral I/R [22], [23]. In focal cerebral I/R models of neonatal rats, the autophagy inhibitor 3-MA provided substantial neuroprotection even when given >4 hours after ischemia [22]. RNAi knockdown of Beclin 1 reduces infarct volume and inhibits histological injury and neurological deficits induced by focal cerebral I/R in adult rats [23], supporting the conclusion that autophagy plays a pro-death role in cerebral I/R. In our study, we also tested the effects of inhibiting autophagy after cerebral I/R. 3-MA treatment before reperfusion significantly reduce the infarct volume. Thus, neuronal autophagy may be a promising therapeutic target for cerebral I/R treatment.

PPAR-γ activation has recently been shown to be a rational and effective strategy against cerebral I/R injury [5]–[8]. PPAR-γ agonists efficiently protect against cerebral I/R in rats. The mechanisms of neuroprotection following PPAR-γ activation include antioxidative properties and anti-inflammatory effect. Recent studies suggest that ligand-activated PPAR-γ controls apoptosis and contributes to neuroprotection [9], [10], [33], [34]. However, whether PPAR-γ activation mediates neuronal autophagy after I/R remains unclear. Therefore, we explore the role of PPAR-γ agonist on neuronal autophagy. Our results showed that cerebral I/R injury enhances the expression of PPAR-γ protein in cortex, maximal levels are observed after 24 h. Treatment with PPAR-γ agonist 15-PGJ_2_ significantly decreased LC3-II, Beclin 1, cathepisin-B, and LAMP1 expression at 12 h after I/R in a concentration-dependant manner. Furthermore, immunohistochemistry analysis showed that 15-PGJ_2_ inhibits intensity of LC3 and cathepsin-B staining in neurons at 12 h after I/R. These results indicated that PPAR-γ agonist 15d-PGJ_2_ inhibits neuronal autophagy after cerebral I/R injury. Moreover, we also showed that inhibition of the autophagic pathway might play a role in 15d-PGJ_2_ neuroprotective effects against cerebral I/R injury.

The mechanisms underlying PPAR-γ agonists mediating neuronal autophagy after cerebral I/R is unclear. Previous studies have demonstrated that ischemia stimulates autophagy through the AMPK-mTOR pathway [35], whereas I/R stimulates autophagy through a Beclin 1-dependent but AMPK-independent pathway [36]. A putative PPAR response element (PPRE) has been reported in the 3′-untranslated region of the Bcl-2 gene in human cancer cells. Moreover, PPAR-γ agonists protect neurons against ischemia/reperfusion damage by enhancing Bcl-2/Bcl-xl [33], [34]. As the autophagy-inducing activity of Beclin 1 is inhibited by multidomain proteins of the Bcl-2 family including Bcl-2, Bcl-xl and Mcl-1 [37]–[39], we hypothesize that PPAR-γ activation might upregulate Bcl-2/Bcl-xl which interact with Beclin 1 and functionally antagonize Beclin 1-mediated autophagy.

In conclusion, our present study demonstrates that PPAR-γ agonist 15d-PGJ_2_ exerts neuroprotection by inhibiting neuronal autophagy after against cerebral I/R injury. Although the molecular mechanisms underlying PPAR-γ agonist in mediating neuronal autophagy remain to be determined, neuronal autophagy may be a new target for PPAR-γ agonist treatment in cerebral I/R injury.

## References

[1] Forman BM, Tontonoz P, Chen J, Brun RP, Spiegelman BM, et al (1995) 15-Deoxy-delta 12, 14-prostaglandin J2 is a ligand for the adipocyte determination factor PPAR gamma. Cell 83: 803–812. 8521497 10.1016/0092-8674(95)90193-0

[2] Mclntyre TM, Pontsler AV, Silva AR, St Hilaire A, Xu Y, et al (2003) Identification of an intracellular receptor for lysophosphatidic acid (LPA): LPA is a transcellular PPARgamma agonist. Proc Natl Acad Sci USA 100: 131–136. 12502787 10.1073/pnas.0135855100 PMC140905

[3] Schopfer FJ, Lin Y, Baker PR, Cui T, Garcia-Barrio M, et al (2005) Nitrolinoleic acid: an endogenous peroxisome proliferator-activated receptor gammar ligand. Proc Natl Acad Sci USA 102: 2340–2345. 15701701 10.1073/pnas.0408384102 PMC548962

[4] Lehmann JM, Moore LB, Smith-Oliver TA, Wikison WO, Willson TM, et al (1995) An antidiabetic thiazolidinedione is a high affinity ligand for peroxiome proliferator-activated receptor gamma (PPARgamma). J Biol Chem 270: 12953–12956. 7768881 10.1074/jbc.270.22.12953

[5] Shimazu T, Inoue I, Araki N, Asano Y, Sawada M, et al (2005) A peroxisome proliferator-activated receptor-γ agonist reduces infarct size in transient but not in permanent ischemia. Stroke 36: 353–359. 15618443 10.1161/01.STR.0000152271.21943.a2

[6] Sundararajan S, Gamboa JL, Victor NA, Wandri EW, Lust WD, et al (2005) Peroxisome proliferator-activated receptor-gamma ligands reduce inflammation and infarction size in transient focal ischemia. Neuroscience 2130: 685–696. 10.1016/j.neuroscience.2004.10.021 15590152

[7] Collino M, Aragno M, Mastrocola R, Gallicchio M, Rosa AC, et al (2006) Modulation of the oxidative stress and inflammatory response by PPAR-γ agonists in the hippocampus of rats exposed to cerebral ischemia/reperfusion. Eur J Pharmacol 530: 70–80. 16386242 10.1016/j.ejphar.2005.11.049

[8] Zhao Y, Patzer A, Herdegen T, Gohlke P, Culman J (2006) Activation of cerebral peroxisome proliferator-activated receptors γ promotes neuroprotection by attenuation of neuronal cyclooxygenase-2 overexpression after focal cerebral ischemia in rats. FASEB J 20: 1162–1175. 16770015 10.1096/fj.05-5007com

[9] Lin TN, Cheung WM, Wu JS, Chen JJ, Lin H, et al (2006) 15d-Prostaglandin J_2_ protects brain from ischemia-reperfusion injury. Atheroscler Thromb Vasc Biol 26: 481–487. 10.1161/01.ATV.0000201933.53964.5b 16385084

[10] Wu JS, Cheung WM, Tsai YS, Chen YT, Fong WH, et al (2009) Ligand-activated peroxisome proliferator-activated receptor-gamma protects against ischemic cerebral infarction and neuronal apoptosis by 14-3-3 epsilon upregulation. Circulation 119: 1124–1134. 19221220 10.1161/CIRCULATIONAHA.108.812537 PMC4144045

[11] Zhu C, Wang X, Xu F, Bahr BA, Shibata M, et al (2005) The influence of age on apoptotic and other mechanisms of cell death after cerebral hypoxia-ischemia. Cell Death Differ 12: 162–176. 15592434 10.1038/sj.cdd.4401545

[12] Zhu C, Xu F, Wang X, Shibata M, Uchiyama Y, et al (2006) Different apoptotic mechanisms are activated in male and female brains after neonatal hypoxia-ischaemia. J Neurochem 96: 1016–1027. 16412092 10.1111/j.1471-4159.2005.03639.x

[13] Adhami F, Liao G, Morozov YM, Schloemer A, Schmithorst VJ, et al (2006) Cerebral ischemia-hypoxia induces intravascular coagulation and autophagy. Am J Pathol 169: 566–583. 16877357 10.2353/ajpath.2006.051066 PMC1780162

[14] Koike M, Shibata M, Tadakoshi M, Gotoh K, Komatsu M, et al (2008) Inhibition of autophagy prevents hippocampal pyramidal neuron death after hypoxic-ischemic injury. Am J Pathol 172: 454–469. 18187572 10.2353/ajpath.2008.070876 PMC2312361

[15] Carloni S, Buonocore G, Balduini W (2008) Protective role of autophagy in neonatal hypoxia-ischemia induced brain injury. Neurobiol Dis 32: 329–339. 18760364 10.1016/j.nbd.2008.07.022

[16] Ginet V, Puyal J, Clarke PGH, Truttmann AC (2009) Enhancement of autophagic flux after neonatal cerebral hypoxia-ischemia and its region-specific relationship to apoptotic mechanisms. Am J Pathol 175: 1962–1974. 19815706 10.2353/ajpath.2009.090463 PMC2774060

[17] Nitatori T, Sato N, Waguri S, Karasawa Y, Araki H, et al (1995) Delayed neuronal death in the CA1 pyramidal cell layer of the gerbil hippocampus following transient ischemia is apoptosis. J Neurosci 15: 1001–1011. 7869078 10.1523/JNEUROSCI.15-02-01001.1995 PMC6577848

[18] Liu C, Gao Y, Barrett J, Hu B (2010) Autophagy and protein aggregation after brain ischemia. J Neurochem 115: 68–78. 20633207 10.1111/j.1471-4159.2010.06905.x PMC3518272

[19] Wang JY, Xia Q, Chu KT, Pan J, Sun LN, et al (2011) Severe global cerebral ischemia-induced programmed necrosis of hippocampal CA1 neurons in rat is prevented by 3-methyladenine: a widely used inhibitor of autophagy. J Neuropathol Exp Neurol 70: 314–322. 21412169 10.1097/NEN.0b013e31821352bd

[20] Wen YD, Sheng R, Zhang LS, Han R, Zhang X, et al (2008) Neuronal injury in rat model of permanent focal cerebral ischemia is associated with activation of autophagic and lysosomal pathways. Autophagy 4: 762–769. 18567942 10.4161/auto.6412

[21] Rami A, Langhagen A, Steiger S (2008) Focal cerebral ischemia induces upregulation of Beclin1 and autophagy-like cell death. Neurobiol Dis 29: 132–141. 17936001 10.1016/j.nbd.2007.08.005

[22] Puyal J, Vaslin A, Mottier V, Clarke PGH (2009) Postischemic treatment of neonatal cerebral ischemia should target autophagy. Ann Neurol 66: 378–389. 19551849 10.1002/ana.21714

[23] Zheng YQ, Liu JX, Li XZ, Xu L, Xu YG (2009) RNA interference-mediated downregulation of Beclin1 attenuates cerebral ischemic injury in rats. Acta Pharmocol Sin 30: 919–927. 10.1038/aps.2009.79 PMC4006642 19574998

[24] Zhou J, Zhang W, Liang B, Casimiro MC, Whitaker-Menezes D, et al (2009) PPARgamma activation induces autophagy in breast cancer cells. Int J Biochem Cell Biol 41: 2334–2442. 19563910 10.1016/j.biocel.2009.06.007 PMC2753745

[25] Clark WM, Lessov NS, Dixon MP, Eckenstein F (1997) Monofilament intraluminal middle cerebral artery occlusion in the mouse. Neurol Res 19: 641–648. 9427967 10.1080/01616412.1997.11740874

[26] Maiuri MC, Zalchavar E, Kimchi A, Kroemer G (2007) Self-eating and self-killing: crosstalk between autophagy and apoptosis. Nat Rev Mol Cell 8: 741–752. 10.1038/nrm2239 17717517

[27] Levine B, Klionsky DJ (2004) Development by self-digestion: molecular mechanisms and biological functions of autophagy. Dev Cell 6: 463–477. 15068787 10.1016/s1534-5807(04)00099-1

[28] Klionsky DJ, Emr SD (2000) Autophagy as a regulated pathway of cellular degradation. Science 290: 1717–1721. 11099404 10.1126/science.290.5497.1717 PMC2732363

[29] Wong E, Cuervo AM (2010) Autophagy gone awry in neurodegenerative diseases. Nat Neurosci 13: 805–811. 20581817 10.1038/nn.2575 PMC4038747

[30] Nixon RA (2006) Autophagy in neurodegenerative disease: friend, foe or turncoat? Trends Neurosci 29: 528–535. 16859759 10.1016/j.tins.2006.07.003

[31] Marino G, Madeo F, Kroemer G (2010) Autophagy for tissue homeostasis and neuroprotection. Curr Opin Cell Bio 23: 1–9. 21030235 10.1016/j.ceb.2010.10.001

[32] Banerjee R, Beal MF, Thomas B (2010) Autophagy in neurodegenerative disorders: pathogenic roles and therapeutic implications. Trends Neurosci 33: 541–549. 20947179 10.1016/j.tins.2010.09.001 PMC2981680

[33] Fuenzalida K, Quintanilla R, Romos P, Piderit D, Fuentealba RA, et al (2007) Peroxisome proliferator-activated receptor γ up-regulates the Bcl-2 anti-apoptotic protein in neurons and induces mitochondrial stabilization and protection against oxidative stress and apoptosis. J Biol Chem 282: 37006–37015. 17965419 10.1074/jbc.M700447200

[34] Wu JS, Lin TN, Wu KK (2009) Rosiglitazone and PPAR-gamma overexpression protect mitochondrial membrane potential and prevent apoptosis by upregulating anti-apoptotic Bcl-2 family proteins. J Cell Physiol 220: 58–71. 19229877 10.1002/jcp.21730

[35] Xu F, Gu JH, Qin ZH (2012) Neuronal autophagy in cerebral ischemia. Neurosci Bull 28: 658–666. 22968594 10.1007/s12264-012-1268-9 PMC5561920

[36] Matsui Y, Takagi H, Qu X, Abdellatif M, Sakoda H, et al (2007) Distinct role of autophagy in the heart during ischemia and reperfusion: roles of AMPK and Beclin 1 in mediating autophagy. Circ Res 100: 914–922. 17332429 10.1161/01.RES.0000261924.76669.36

[37] Pattingre S, Tassa A, Qu X, Garuti R, Liang XH, et al (2005) Bcl-2 antiapoptotic proteins inhibit Beclin1-dependent autophagy. Cell 122: 927–939. 16179260 10.1016/j.cell.2005.07.002

[38] He C, Levine B (2010) The Beclin 1 interactome. Curr Opin Cell Biol 22: 140–149. 20097051 10.1016/j.ceb.2010.01.001 PMC2854269

[39] Maiuri MC, Criollo A, Kroemer G (2010) Crosstalk between apoptosis and autophagy within the Beclin 1 interactome. EMBO J 29: 515–516. 20125189 10.1038/emboj.2009.377 PMC2830702

